# Changes in flea and tick infestation following topical fipronil, permethrin, piperonyl butoxide, and pyriproxyfen in naturally infested dogs: an exploratory uncontrolled field study

**DOI:** 10.3389/fvets.2026.1903878

**Published:** 2026-10-13

**Authors:** Camilo Romero-Núñez, Rafael Heredia-Cárdenas, Ariadna Flores-Ortega, Linda Guiliana Bautista-Gómez, Soila Maribel Gaxiola-Camacho, José David Torres-Cruz

**Affiliations:** 1 Hospital Veterinario DERMAVET, Mexico City, Mexico; 2 Centro Integral Veterinario, CIVET, Mexico; 3 Centro Universitario UAEM-Amecameca, Universidad Autónoma del Estado de México, Amecameca, Mexico, Mexico; 4 Facultad de Medicina Veterinaria y Zootecnia, Universidad Autónoma de Sinaloa, Culiacán, Mexico

**Keywords:** dogs, ectoparasite control, fipronil, permethrin, piperonyl butoxide, pyriproxyfen

## Abstract

**Background:**

Flea and tick infestations impair canine welfare and may have public-health relevance in rural settings.

**Objective:**

To describe temporal changes in ectoparasite infestation and associated clinical signs after a single topical application of fipronil, permethrin, piperonyl butoxide, and pyriproxyfen (PROTECH) in naturally infested dogs.

**Methods:**

This prospective, uncontrolled exploratory field study enrolled 44 privately owned dogs in Tahdziú, Yucatán, Mexico. Dogs received one topical application at Day 0 and were reassessed on Days 15 and 30. An approximately 5-min whole-body examination documented ectoparasite presence or absence. Field-recorded numerical skin-lesion and pruritus scores and observed adverse events were summarized.

**Results:**

Among dogs with flea-only infestation (*n* = 18), positivity decreased from 18/18 at Day 0 to 0/18 at Days 15 and 30. Among tick-only dogs (*n* = 11), positivity decreased from 11/11 to 0/11 and 1/11, respectively. In mixed infestations (*n* = 15), positivity decreased from 15/15 to 3/15 and 2/15. Mean lesion and pruritus scores also declined in all subgroups. No adverse reactions were observed.

**Conclusions:**

A single application was followed by marked temporal reductions in ectoparasite positivity and clinical signs through Day 30. Because the study lacked a comparator and used non-validated clinical scores, the findings are descriptive and exploratory; they do not establish a causal treatment effect or comparative efficacy.

## 1 Introduction

Fleas and ticks are common ectoparasites of dogs and can cause pruritus, dermatitis, blood loss, and reduced welfare. They may also carry pathogens of veterinary or zoonotic importance. Sustained ectoparasite control is particularly challenging in rural communities where access to veterinary care and preventive products is limited and dogs remain exposed to untreated animals and contaminated environments (1–7).

Tahdziú, Yucatán, is a pre-dominantly Maya-speaking rural community with limited access to veterinary services and routine ectoparasite prevention (8). These conditions provide a relevant setting in which to document temporal patterns after treatment under natural reinfestation pressure. Nevertheless, an uncontrolled before-and-after evaluation cannot distinguish a treatment-associated change from natural variation, seasonal effects, environmental conditions, owner practices, or regression to the mean. Measuring ectoparasite burden alone also cannot establish an effect on pathogen infection or disease transmission.

The objective of this exploratory field study was to describe changes in flea and tick infestation status, skin-lesion scores, pruritus scores, and observed tolerability during 30 days after a single topical application of fipronil, permethrin, piperonyl butoxide, and pyriproxyfen in naturally infested dogs.

## 2 Materials and methods

Study site and period. The prospective, uncontrolled field study was conducted from March to May 2023 in Tahdziú, Yucatán, Mexico. The community has limited access to veterinary services, and participating dogs generally did not receive routine preventive ectoparasite control (8). Dogs remained in their usual households and environments throughout follow-up; therefore, environmental reinfestation pressure was not controlled or quantified.

### 2.1 Animals

A convenience sample of 44 privately owned dogs was enrolled irrespective of breed, sex, age, or health status. Written informed consent was obtained from all owners after the procedures had been explained. Owners completed a structured epidemiologic questionnaire concerning breed, age, sex, body condition, housing, other household animals, environmental hygiene, previous antiparasitic use, veterinary visits, and vaccination history. No *a priori* sample-size or power calculation was performed; enrollment was determined by the dogs available during the community campaign. The resulting flea-only (*n* = 18), tick-only (*n* = 11), and mixed-infestation (*n* = 15) subgroups were therefore considered exploratory.

At Day 0, Day 15, and Day 30, each dog underwent an approximately 5-min whole-body visual and manual examination under gloved restraint by owners and veterinarians. The coat and skin were inspected systematically over the head and ears, neck, dorsal and ventral trunk, axillary and inguinal regions, limbs, interdigital areas, and tail region; visible ectoparasites were recorded as present or absent. The same examination duration was used for all dogs to standardize search effort. Ectoparasites were classified morphologically using published taxonomic keys (9, 10). The original field records did not retain quantitative parasite counts or a species-level distribution suitable for analysis; consequently, results are reported by infestation category rather than parasite burden or species. Skin lesions and pruritus were recorded as numerical field scores at each visit. The surviving study records contain the aggregated mean scores but not the original scoring rubric, item-level criteria, or individual scores. These measures were not based on formally validated dermatological or pruritus instruments. Evaluators were not blinded, and neither evaluator training nor interobserver agreement was documented. The clinical scores are therefore presented only as descriptive exploratory outcomes. All dogs remained under their customary feeding and husbandry practices and in their naturally exposed environments.

### 2.2 Treatment

At Day 0, all ectoparasite-positive dogs received a single topical spot-on application of PROTECH, containing fipronil 10.0 g, permethrin 40.0 g, piperonyl butoxide 3.0 g, and pyriproxyfen 10.0 g, with excipients q.s. to 100 ml (PROTECH, México, S.A. de C.V., Mexico). The product was applied directly to the skin along the dorsal midline between the interscapular region and the base of the ears according to body weight and label instructions.

### 2.3 Statistical analysis

Analyses were exploratory. Baseline characteristics were summarized descriptively. Because parasite status was repeatedly measured in the same dogs, baseline-vs.-follow-up binary comparisons were reviewed using two-sided exact McNemar tests rather than Fisher's exact test. These tests could be reconstructed from the reported paired totals because every dog was positive at baseline and follow-up was complete. Skin-lesion and pruritus outcomes are reported only as aggregated means and percentage changes over time. Although the original report contained *p*-values generated in the JMP Matched Pairs platform, those *p*-values were removed because the individual-level dataset and exact non-parametric procedure are no longer available for independent reconstruction. No multiplicity adjustment was pre-specified. No observations were reported missing. Reproducible analyses of parasite positivity were performed using the paired totals reported in the article; the original analyses were conducted in JMP version 8.0 (SAS Institute Inc., Cary, NC, USA).

## 3 Results

A total of 44 dogs were enrolled and completed all three evaluations: 27 males and 17 females; 42 mixed-breed dogs, one Dachshund, and one Labrador Retriever. Thirteen were classified as large, 28 as medium, and three as small. Mean age was 3.3 ± 2.5 years. No adverse reactions were observed following treatment. Associations between baseline infestation and demographic variables were not emphasized because of the small subgroup sizes and exploratory design.

In the flea-only subgroup, positivity declined from 18/18 dogs (100%; 95% CI, 82.4–100) at Day 0 to 0/18 (0%; 95% CI, 0–17.6) at both Day 15 and Day 30 (exact McNemar *p* < 0.001 for each comparison with Day 0). Mean skin-lesion scores declined by 63.3% at Day 15 and 88.8% at Day 30 relative to baseline. Mean pruritus scores declined by 39.9% and 98.1%, respectively (Table 1). Clinical-score changes are descriptive because individual-level observations were unavailable for verification.

**Table 1:** Flea positivity, skin-lesion scores, and pruritus scores after PROTECH treatment (*n* = 18).

Outcome	Day 0	Day 15	Day 30	*P*: D0 vs D15	*P*: D0 vs D30
Flea-positive	18/18 (100%; 95% CI 82.4–100)	0/18 (0%; 95% CI 0–17.6)	0/18 (0%; 95% CI 0–17.6)	< 0.001^*^	< 0.001^*^
Skin-lesion score, mean	271.83	99.77 (−63.3%)	30.33 (−88.8%)	Not reported	Not reported
Pruritus score, mean	5.72	3.44 (−39.9%)	0.11 (−98.1%)	Not reported	Not reported

^*^Two-sided exact McNemar test, reconstructed from paired totals. Clinical-score p-values are not reported because the individual-level data and exact original procedure were unavailable for independent verification. Percentages in parentheses indicate descriptive reduction from Day 0. Confidence intervals for proportions are Wilson 95% intervals.

In the tick-only subgroup, positivity declined from 11/11 dogs (100%; 95% CI, 74.1–100) at Day 0 to 0/11 (0%; 95% CI, 0–25.9) at Day 15 and 1/11 (9.1%; 95% CI, 1.6–37.7) at Day 30. Exact McNemar comparisons with Day 0 yielded *p* < 0.001 at Day 15 and *p* = 0.002 at Day 30. The Day 30 positive finding may represent reinfestation or persistence in one dog. Mean skin-lesion scores declined by 61.5 and 89.1%, and mean pruritus scores by 59.7 and 80.6%, at Days 15 and 30, respectively (Table 2). These score changes are descriptive.

**Table 2:** Tick positivity, skin-lesion scores, and pruritus scores after PROTECH treatment (*n* = 11).

Outcome	Day 0	Day 15	Day 30	*P*: D0 vs D15	*P*: D0 vs D30
Tick-positive	11/11 (100%; 95% CI 74.1–100)	0/11 (0%; 95% CI 0–25.9)	1/11 (9.1%; 95% CI 1.6–37.7)	< 0.001^*^	0.002^*^
Skin-lesion score, mean	159.18	61.36 (−61.5%)	17.36 (−89.1%)	Not reported	Not reported
Pruritus score, mean	5.63	2.27 (−59.7%)	1.09 (−80.6%)	Not reported	Not reported

^*^Two-sided exact McNemar test, reconstructed from paired totals. Clinical-score p-values are not reported because the individual-level data and exact original procedure were unavailable for independent verification. Percentages in parentheses indicate descriptive reduction from Day 0. Confidence intervals for proportions are Wilson 95% intervals.

In the mixed-infestation subgroup, positivity declined from 15/15 dogs (100%; 95% CI, 79.6–100) at Day 0 to 3/15 (20.0%; 95% CI, 7.1–45.2) at Day 15 and 2/15 (13.3%; 95% CI, 3.7–37.9) at Day 30. Exact McNemar comparisons with Day 0 yielded *p* < 0.001 at both follow-ups. Mean skin-lesion scores declined by 56.2% at Day 15 and 81.5% at Day 30 (Table 3). These score changes are descriptive.

**Table 3:** Mixed-infestation positivity, skin-lesion scores, and pruritus scores after PROTECH treatment (*n* = 15).

Outcome	Day 0	Day 15	Day 30	*P*: D0 vs D15	*P*: D0 vs D30
Mixed-infestation positive	15/15 (100%; 95% CI 79.6–100)	3/15 (20.0%; 95% CI 7.1–45.2)	2/15 (13.3%; 95% CI 3.7–37.9)	< 0.001^*^	< 0.001^*^
Skin-lesion score, mean	200.93	88.06 (−56.2%)	37.13 (−81.5%)	Not reported	Not reported
Pruritus score, mean	6.80	3.66 (−46.2%)	1.93 (−71.6%)	Not reported	Not reported

^*^Two-sided exact McNemar test, reconstructed from paired totals. Clinical-score p-values are not reported because the individual-level data and exact original procedure were unavailable for independent verification. Percentages in parentheses indicate descriptive reduction from Day 0. Confidence intervals for proportions are Wilson 95% intervals.

Mean pruritus scores in the mixed-infestation subgroup declined by 46.2% at Day 15 and 71.6% at Day 30 relative to baseline. Because no untreated or active-comparator group was included, these temporal improvements cannot be attributed exclusively to the product.

## 4 Discussion

This exploratory uncontrolled field study documented marked temporal reductions in flea and tick positivity and associated clinical signs after a single topical application. The observations were made while dogs remained in their usual rural environments. However, the before-and-after design does not establish that PROTECH caused the changes, and the findings should not be interpreted as proof of efficacy.

The temporal pattern observed here is compatible with reports from controlled studies of fipronil-permethrin formulations (11, 12), but it is not directly comparable with estimates from those designs. In the present study, all flea-only dogs were negative at Days 15 and 30; all tick-only dogs were negative at Day 15, while one dog was positive at Day 30. The latter observation is compatible with ongoing environmental exposure, reinfestation, or persistence. Because environmental parasite abundance, household treatment, weather, contact with untreated animals, owner practices, and seasonal change were not measured, their contributions cannot be separated from the post-treatment pattern.

No adverse reactions were observed during the 30-day field follow-up. This finding supports short-term observed tolerability in the enrolled dogs, but the sample was too small to exclude uncommon adverse events and no untreated comparator was available.

Aggregated lesion and pruritus scores declined alongside ectoparasite positivity. This temporal relationship is clinically plausible because flea and tick exposure can contribute to inflammatory and hypersensitivity-associated skin disease (13–16). However, the scoring rubric and individual observations were not retained, the instruments were not validated, evaluators were not blinded, and interobserver reliability was not measured. The original score-related *p*-values were therefore removed, and the remaining mean values and percentage changes should be interpreted only as descriptive exploratory observations.

The formulation combines adulticidal compounds with piperonyl butoxide and the insect growth regulator pyriproxyfen (17). These mechanisms provide biological plausibility for the observed reductions. Mechanistic plausibility, however, does not replace the need for a controlled comparison, and the present study was not designed to estimate the independent contribution of each active ingredient.

Fleas and ticks can carry pathogens of veterinary and zoonotic importance, including Rickettsia and Bartonella species (18–28). The present study did not test ectoparasites, dogs, or humans for pathogens and did not measure vector infection, exposure, or transmission outcomes. Therefore, reduced zoonotic risk remains a potential implication of effective ectoparasite-control programs, not an outcome demonstrated by this study.

Community-based ectoparasite-control programs may improve canine welfare in underserved settings, particularly when treatment is combined with owner education, repeated prevention, treatment of in-contact animals, and environmental management (23, 28–33). Future studies should include untreated or active comparator groups, pre-specified sample-size calculations, standardized quantitative parasite counts, species-level identification, validated clinical scales, blinded trained evaluators, interobserver-reliability assessment, secure retention of de-identified individual-level data, and pathogen outcomes if One Health effects are to be tested.

### 4.1 Limitations

The study had no negative-control or active-comparator group, used a convenience sample without an *a priori* power calculation, and included small infestation subgroups. Seasonal change, natural variation, regression to the mean, owner behavior, and unmeasured environmental reinfestation pressure may have contributed to the observed changes. Parasite burden was recorded as presence or absence after a time-limited examination; quantitative counts and species-level distributions were not retained. The lesion and pruritus measures were non-validated field scores, and their original rubric, individual observations, evaluator blinding, and interobserver reliability were unavailable. The individual-level file was not preserved during data archiving after the original analysis; only summarized tables and software outputs remain. Consequently, the score analyses cannot be independently reconstructed. These limitations substantially restrict causal inference, reproducibility, and generalizability.

## 5 Conclusion

A single topical application of PROTECH was followed by marked temporal reductions in flea and tick positivity and by lower aggregated skin-lesion and pruritus scores through Day 30 in naturally infested dogs. No adverse reactions were observed. These uncontrolled before-and-after observations are preliminary and descriptive; they do not establish that the product caused the reductions or demonstrate comparative efficacy. Controlled studies using validated outcomes and reproducible individual-level data are required.

## Data Availability

The data supporting the findings of this study are available from the corresponding author upon reasonable request.
